# Comparison of total disc arthroplasty and fusion in treatment of lumbar disc disease: A cohort study protocol

**DOI:** 10.1097/MD.0000000000022024

**Published:** 2020-08-28

**Authors:** Yi Wang, Yunting Bai, Haoguang Ma, Shaolei Wang

**Affiliations:** a Department of Orthopaedics, People's Hospital of Mianzhu, Mianzhu 618200, Sichuan; b Department of Orthopaedics, The Fifth People's Hospital of Jinan, Jinan 250022; c Department of Surgery, Hot Spring Sanatorium of Linyi, Shandong Coal (Linyi Hedong Central Hospital), Linyi 276032, Shandong; d Department of Orthopaedics, Shaoxing People's Hospital (Shaoxing Hospital of Zhejiang University School of Medicine), Shaoxing 312000, Zhejiang, China.

**Keywords:** lumbar disc disease, protocol, spinal fusion, total disc arthroplasty

## Abstract

**Background:**

In recent years, the clinical efficacy of spinal fusion (SF) or total disc arthroplasty (TDA) in the treatment of the degenerative lumbar disc disease is still controversial. The objective of this retrospective clinical trial was to investigate whether TDA was superior to the SF in the complication rates and clinical outcome scores.

**Methods:**

This retrospective research was based on the Strengthening the Reporting of Observational studies in Epidemiology checklist. Internal clinical data sets for 2014 to 2018 were acquired and consolidated with the approval of the Institutional Review Committee of Shaoxing Hospital of Zhejiang University. Inclusion criteria in this present research included: low back pain without or with the leg pain for more than one year; failure of conservative treatment planned for more than three months; age was 25 to 60 years old; followed up for at least one year. The main outcome measure was disability and pain measured via the Norwegian version of Oswestry disability index 2.0. The other clinical outcomes included Short-Form Health Survey, reoperations, duration of surgery, complications, hospital stay length, as well as the blood loss. The significance was set at 0.05 level with the confidence intervals of 95%. The software package of SPSS (version 21.0; SPSS Inc, Chicago, IL, USA) was applied for all the analyses of statistics.

**Results:**

The null hypothesis is that there is no significant difference in outcomes between TDA and SF in the treatment of degenerative lumbar disc disease.

**Trial registration:**

This study protocol was registered in Research Registry (researchregistry5847).

## 1 Introduction

Low back pain is thought to be the leading cause of disability worldwide, with an estimated 632 million people affected.^[1,2]^ The resulting social burden is serious. It is estimated that the socio-economic of the United States exceed 100 billion dollars a year.^[3,4]^ Lumbar disc disease is the main cause of low back pain. It is the major reason of low back pain and is related to environmental and genetic factors.^[5–7]^ With the progressive degeneration of intervertebral disc, the effectiveness of intervertebral disc nutrition mechanism is reduced, and nucleus pulposus cells lose their ability to generate extracellular matrix proteoglycan and proteins, which leads to progressive instability and dryness of intervertebral disc.^[8]^

Lumbar spinal fusion (SF) has been regarded as a gold standard for the surgical treatment of the degenerative disc disease. If the patients with debilitating back pain cannot be relieved by non-surgical treatment, and the source of the pain is considered to be located in the motor segment, surgical fusion can be considered to eliminate the painful movement.^[9–11]^ Nevertheless, the decrease of segment mobility also increases the stress of adjacent segments, especially the neighboring segment. This may cause the recurrence of symptoms, i.e. neighboring segment disease, and requires a in-depth surgery.^[12]^ In order to restore the motion of the spine and overcome the SF surgery shortcomings, it is assumed that lumbar total disc arthroplasty (TDA) was utilized to restore the intervertebral disc function. It is a replacement for SF in the carefully selected patients with symptomatic degenerative disc disease and has been utilized for more than 13 years. Cochrane review found that the TDA has a statistically significant over the SF, but this difference was not clinically significant.^[13]^

In recent years, the clinical efficacy of SF or TDA in the treatment of the degenerative lumbar disc disease is still controversial. The objective of this retrospective clinical trial was to investigate whether TDA was superior to the SF in the complication rates and clinical outcome scores. The null hypothesis is that there is no significant difference in outcomes between TDA and SF in the treatment of degenerative lumbar disc disease.

## 2 Materials and methods

### 2.1 Study design

This retrospective research was based on the Strengthening the Reporting of Observational studies in Epidemiology checklist. Internal clinical data sets for 2014 to 2018 were acquired and consolidated with the approval of the Institutional Review Committee of Shaoxing Hospital, School of Medicine, Zhejiang University (ZJSX10740). Our research was registered with Research Registry (researchregistry5847).

### 2.2 Inclusion and exclusion criteria

Inclusion criteria in this present research included: low back pain without or with the leg pain for more than one year; failure of conservative treatment planned for more than three months; age was 25 to 60 years old; followed up for at least one year. Exclusion criteria included: spinal stenosis requires the decompression; pain level 3 or above at the clinical examination; isthmic spondylolisthesis or spondylolysis; former spinal tumor or infection; and the inability to understand information owing to medical, psychological or the abuse reasons.

### 2.3 Surgical techniques

Patients in this current work received either a retroperitoneal or transperitoneal approach to the lumbosacral vertebra and underwent the full anterior discectomy. The cartilage endplates and nucleus pulposus were removed after thoracolumbar discectomy, while the bone endplates were preserved.

#### 2.3.1 TDA technique

In group TDA, with the exception of the lateral ring, complete discectomy was performed to disperse the intervertebral disc space and release the posterior longitudinal ligament to ensure the segmental activity. The cartilaginous endplates were removed carefully using the curettes to keep the subchondral bony endplates integrity. The insertion procedure, TDA size as well as ultimate end plate preparation were in accordance with the respective recommendations of manufacturer.

#### 2.3.2 SF technique

In group SF, according to the operation habits, the fusion mode was posterior lumbar interbody fusion or posterolateral fusion, internal fixation with pedicle screws, Monarch instrumentation or Steffee plates was used (DePuy Spine). Autologous bone graft, locally or from the posterior iliac crest, was used.

### 2.4 Outcome evaluation

Preoperative characteristics: American Society of Anesthesiologists (ASA) grade, male, age, weight and height, duration of surgery, smoking, hypertension, length of stay, as well as amount of blood loss were directly harvested from our hospital database. ASA grade ≥3 points indicated obvious systemic disease. The body mass index (BMI) was calculated according to weight and height. The main outcome measure was disability and pain measured via the Norwegian version of Oswestry disability index 2.0 (scores between 0 and 100, and the lower the score, the less disability and pain). In our study, other clinical outcomes included Short-Form Health Survey, reoperations, duration of surgery, complications, hospital stay length, as well as the blood loss (Tables 1 and 2).

**Table 1:**
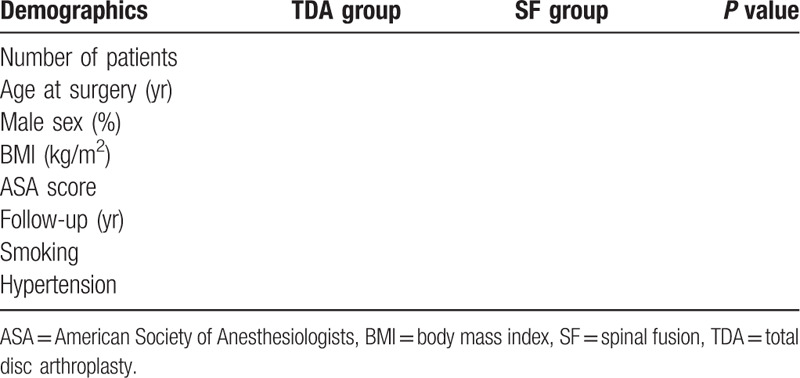
Patient baseline demographics.

**Table 2:**
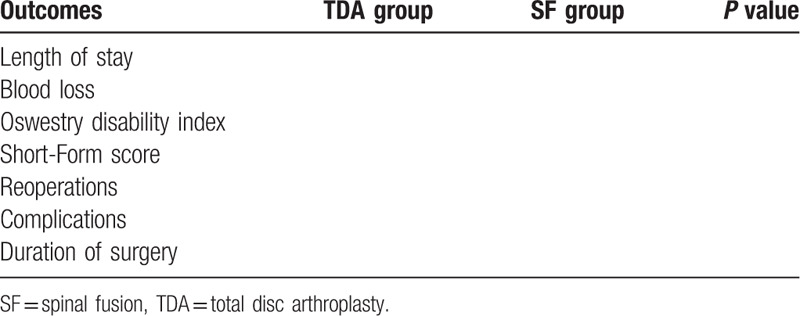
Postoperative outcomes.

### 2.5 Statistical analysis

Age, Oswestry disability index and BMI, Short-Form Health Survey, duration of surgery, hospital stay length, as well as the amount of bleeding were compared between these two groups using Mann-Whitney *U* test and Student *t* test. Chi-square test and the Fisher's exact test were utilized to compare the smoking, hypertension, gender, reoperation and complications between these two groups. The significance was set at 0.05 level with the confidence intervals of 95%. The software package of SPSS (version 21.0; SPSS Inc, Chicago, IL, USA) was applied for all the analyses of statistics.

## 3 Discussion

This retrospective clinical trial was designed to address the issue of whether TDA is superior to the SF in terms of complication rates and clinical outcome scores. The null hypotheses were that TDA does not differ obviously from the SF in the treatment of degenerative lumbar disc disease.

The limitations of our current work included a single surgeon's practice, the lack of patient randomization, and the use of a single implant model, as well as a single implant manufacturer. Furthermore, the limitations of this research include those inherent in any retrospective cohort study, including the observation bias or the possibility of selection.

## Author contributions

**Conceptualization:** Haoguang Ma.

**Data curation:** Yi Wang, Yunting Bai.

**Formal analysis:** Yi Wang, Yunting Bai.

**Funding acquisition:** Shaolei Wang.

**Investigation:** Yi Wang, Yunting Bai.

**Methodology:** Haoguang Ma.

**Resources:** Shaolei Wang, Haoguang Ma.

**Supervision:** Shaolei Wang.

**Writing – original draft:** Yi Wang, Yunting Bai.

**Writing – review & editing:** Shaolei Wang, Haoguang Ma.
